# Agriculture increases potential health risks of vertebrate viruses in soils

**DOI:** 10.1002/imt2.70034

**Published:** 2025-04-16

**Authors:** Kankan Zhao, Yiling Wang, Ran Xue, Xingmei Liu, Bin Ma, Jianming Xu

**Affiliations:** ^1^ State Key Laboratory of Soil Pollution Control and Safety Zhejiang University Hangzhou China; ^2^ Zhejiang Provincial Key Laboratory of Agricultural Resources and Environment Zhejiang University Hangzhou China; ^3^ Hangzhou Global Scientific and Technological Innovation Center, Zhejiang University Hangzhou China

## Abstract

Here, we conducted a large‐scale investigation of vertebrate viruses in soils and found soil was a mediator of vertebrate viruses. Compared to natural soils, agricultural soils possessed distinct prevalence patterns, with a higher detection rate and richness for vertebrate viruses and higher potential health risks.

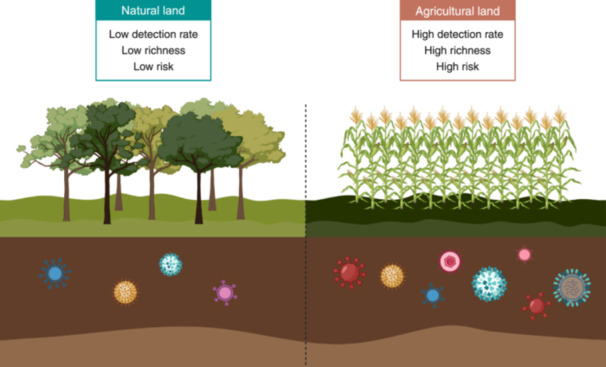

To the Editor,

Vertebrate viruses can adversely affect the health of humans, livestock, and wild animals, triggering broader socioeconomic consequences directly and indirectly. The increasing frequency of pandemics of vertebrate viruses causes severe global distress and increases public attention on One Health. Unlike traditional approaches that focus on host health in isolation, One Health emphasizes the roles of intrinsic interconnections between hosts and their shared environment in achieving optimal health outcomes. Hence, as the foundation of terrestrial organisms and the critical interface of their interactions, soils are regarded as a cornerstone of One Health [1].

Although soils harbor the most diverse and complex microbiome on Earth, the occurrence of vertebrate viruses in soils remains unknown. Vertebrate viruses can be transferred from hosts to soils via wastewater, sewage, manure, dead bodies, etc. Soils provide protection from UV radiation to vertebrate viruses, which can exist in soils for days to months or longer depending on soil properties such as pH, temperature, moisture, organic matters, clay content, and cation exchange capacity [2]. Additionally, characteristics (e.g., presence/absence of an envelope and ability to aggregate) of vertebrate viruses and soil microbial activities would also affect their persistence in soils [3]. In turn, vertebrate viruses can enter hosts by direct contact with soils or indirect contact with vectors, food webs, aerosols, and so on. Therefore, understanding the occurrence of vertebrate viruses in soils is essential to develop effective strategies for managing their risks to public health.

Agricultural expansion and intensification, affecting many biotic and abiotic processes in soil ecosystems, are important mediators of vertebrate virus pandemics [4]. It encroaches into wildlife habitats, expands the prevalence and host range of vertebrate viruses [5], strengthens interaction frequency and intensity between vertebrate viruses, hosts, and environments [6], accelerates the evolution of resistance and virulence of vertebrate viruses [7], and thus increases exposure possibilities and health risks of hosts to vertebrate viruses. Meanwhile, agriculture‐related activities are also key to tackling global sustainability challenges such as population explosion, climate change, and food security, which in turn would gain new urgency in agricultural expansion and intensification [8]. It is, therefore, critical to assess the effects of agriculture on the prevalence and health risks of vertebrate viruses in soils.

While the detection of viral fragments and transcripts in soils may not imply the infectivity of vertebrate viruses to their hosts, metagenome (MG) and metatranscriptome (MT) approaches can still record their prevalence. Hence, to investigate prevalence patterns of vertebrate viruses in soils at global scale, we conducted an integrative analysis with 3049 soil MG samples and 1441 soil MT samples (Figure S1 and Table S1) against a manually curated database of vertebrate viruses (Table S2). We applied a macro‐ecological framework to reveal their occurrence states in soils and the governing mechanisms and quantitatively evaluated their potential health risks in soils. Our study demonstrated the prevalence of vertebrate viruses in soils and revealed their increasing potential health risks in agricultural soils.

## RESULTS AND DISCUSSION

### Soil serves as a mediator for vertebrate viruses

**Figure 1:**
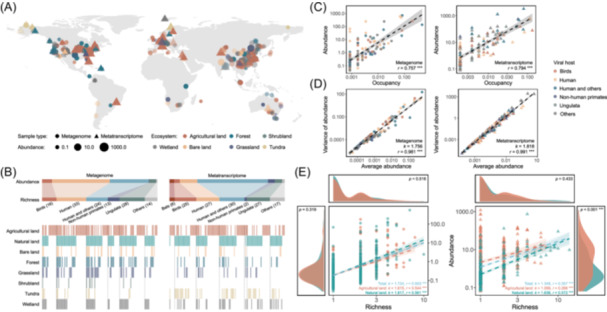
Prevalence patterns of vertebrate viruses. (A) Geographic distribution of metagenome (circles) and metatranscriptome (triangles) samples (colored by ecosystems and sized by abundance) detected vertebrate viruses. (B) The upper panels show the proportions of vertebrate viruses (colored by host groups) in abundance and richness among metagenome and metatranscriptome samples (number represents richness). The lower panels show ecosystems that detected vertebrate viruses corresponding to the upper panels (colors represent vertebrate viruses that existed in the soils of certain ecosystems; nonagricultural ecosystems were grouped into “Natural land”). (C) Relationships between total abundance and occupancy in metagenome (circles) and metatranscriptome (triangles) samples. (D) Relationships between the variance of abundance and average abundance in metagenome (circles) and metatranscriptome (triangles) samples. For (C) and (D), the dash lines are regression lines, and the shaded area is the 95% confidence interval. Colors represent host groups; *k* represents the exponent of Taylor's law and symbol *** represents *p* < 0.001; *r* represents Pearson correlation coefficient. (E) Relationships between abundance and richness of vertebrate viruses. The dash lines are regression lines, and the shaded area is the 95% confidence interval. Colors represent ecosystems, *k* represents the slope of regression line, *r* represents the Pearson correlation coefficient, and symbol *** represents *p* < 0.001. Curvilinear polygons show estimations of frequency densities of richness and abundance.

Our study identified 128 distinct vertebrate viruses from 38.6% of MG samples (1177/3049) and 134 distinct vertebrate viruses from 36.6% of MT samples (528/1441) (Figures 1A and S2A, Tables S3 and S4). The abundance of each vertebrate virus within individual samples exhibited a notably positive correlation in the results of BLASTn and Kraken2 (Figure S3A,B and Table S5), suggesting their results were comparable and consistent. Lower abundance in the results of BLASTn (Figure S3C,D) suggests its parameters were stringent to detect vertebrate virus reads. The rarefaction curves indicate most vertebrate viruses in MG and MT samples had been detected (Figure S4). Besides, there was no significant linear relationship between the richness and sample size of MG and MT samples (Figure S5), indicating the richness of vertebrate viruses did not synchronize with sampling range expansion (i.e., sequencing depth increasing).

Richness and abundance within soil samples varied across host group and family levels of vertebrate viruses (Figures 1B and S2B). Although human‐related (human‐only and zoonotic) viruses were detected in less than half of richness in MG (44.5%) and MT (42.5%) samples, they occupied 84.2% and 76.7% of abundance, respectively.

### Most vertebrate viruses are conditionally rare

Occupancy of vertebrate viruses in soils followed a power‐law distribution with different parameters for different ecosystems (Figure S6). This clearly showed that most vertebrate viruses had low occupancy in soils, with the majority classified as rare species using a criterion of <0.1% occupancy (66.4% in MG and 54.5% in MT samples). These power‐law occupancy frequency distributions were expected under the assumption that gains and losses of vertebrate viruses in soil samples were nearly neutral, leading to low occupancy of the majority of vertebrate viruses in soils [9].

The occupancy‐abundance relationships of vertebrate viruses were significantly positively correlated in MG and MT samples (Figure 1C and Table S3), representing a gradient ranging from a restricted spatial distribution with low abundance (bottom left) to ubiquitous distribution with high abundance (top right) [10]. This trend has been widely observed across various macro‐ and micro‐organisms, leading to the concept of conditionally rare taxa that the “bottom left” species have the potential to bloom [10]. However, the underlying mechanisms of conditionally rare taxa are still under debate, which brings challenges in preventing or mitigating vertebrate virus pandemics.

To further understand the positive correlation between occupancy and abundance of vertebrate viruses in soils, we linked their average abundances (*M*) with spatial variances of abundance (*V*), which was well‐described by Taylor's law (Figure 1D), that is, *V* = *aM* ^*k*^ (*a* is a constant, *k* is the exponent of *M*), implying a dependency between average and variance of abundance across soil samples. The average abundance alone can characterize the distribution of abundance fluctuations [11] for each vertebrate virus within soils. In principle, the exponent *k* ranged between 1 and 2, where the exponent *k* of 2 can be due to demographic and environmental stochasticity (i.e., stochastic fluctuations in abundance and environment) [12], while the exponent *k* less than 2 can result from indirect interactions (i.e., competition through shared hosts) [13]. Combined with the properties of conditionally rare taxa, it is important to implement both offensive and defensive strategies. High‐abundance areas of vertebrate viruses with high average abundances should be targeted with offensive strategies to limit their dispersal. Meanwhile, defensive efforts should be employed to reduce the vulnerability of low‐abundance areas to colonization [14].

### Agriculture alters prevalence patterns of vertebrate viruses in soils

The prevalence patterns of vertebrate viruses across soil samples had strong ecosystem specificity. The detection rate of vertebrate viruses in agricultural soils was much higher than in natural soils in both MG (47.2% vs. 33.8%) and MT (42.0% vs. 30.4%) samples. Moreover, vertebrate viruses detected in multiple ecosystems may indicate their ability to be transferred across ecosystem boundaries. Generally, vertebrate viruses occurred more frequently in agricultural soils than in natural soils, with only 37.5% (48/128) and 23.9% (32/134) of vertebrate viruses shared between agricultural and natural soils in MG and MT samples, respectively (Figures 1B and S2B). Moreover, agricultural soils possessed a higher predicted total richness of vertebrate viruses (Figure S7), indicating agriculture may expand the richness for vertebrate viruses in soils.

We then tested the log–log relationships between abundance and richness to disentangle coexistence patterns and mechanisms of vertebrate viruses in certain ecosystems (Figure 1E). Vertebrate viruses in soils are generally recruited from outside but not inside, which indicates they are typically introduced from external sources. The positive and significant linear relationships are consistent with the hypothesis that richness is controlled by migration (the available species pool) [15], also indicating soil is an important mediator of vertebrate viruses. Furthermore, these observed positive linear relationships demonstrate the presence of vertebrate viruses in soils spans a spectrum from one extreme to another. At one end of the spectrum, low richness with low abundance suggests low rates of colonization, population densities, and interaction intensities, which are opposite at the other extreme [16].

Remarkably, the richness of vertebrate viruses in agricultural soils possessed weaker linear correlations with abundance than in natural soils, especially in MT samples (Figures 1E and S8). Instead, the richness of vertebrate viruses in agricultural soils formed a hump‐shaped relationship. Although agricultural soils had higher richness of vertebrate viruses, the peak of the curve suggests the richness of an individual agricultural soil sample was lower than that of natural soil sample. This intriguing relationship can be explained by the intermediate‐disturbance hypothesis, which postulates that diversity peaks at an intermediate level of abundance and disturbance [16]. At either end of the spectrum, where abundance is low or disturbance is high, only a limited number of opportunistic species can exist. Conversely, when abundance is high or disturbance is low, only a few top competitors persist. The lower richness observed in high‐abundance conditions within agricultural soils may be attributed to anthropogenic disturbance, as few species in the local pool can be adapted to these conditions [17]. Given that most vertebrate viruses can colonize and go extinct stochastically, they may immigrate from distant pools and simply replace the existing ones by increasing abundance [17], complicating the coexistence of viruses in soils.

### Agriculture increases the potential health risks of vertebrate viruses in soils

**Figure 2:**
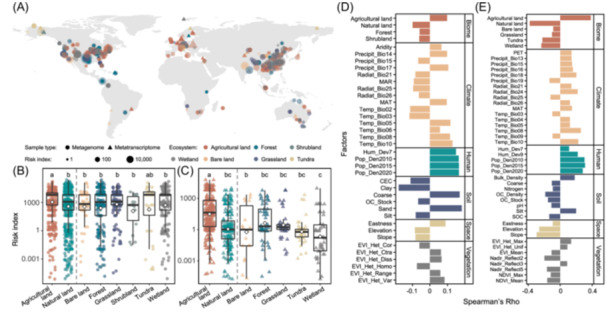
Risk evaluation of vertebrate viruses. (A) The risk index of globally distributed metagenome (circles) and metatranscriptome (triangles) samples colored by ecosystems and sized by the risk index of a sample. Risk index of metagenome (B) and metatranscriptome (C) samples across ecosystems. Boxes are vertically bounded by the 1st and 3rd quartiles, center lines are medians, diamonds are means, and whiskers extend to ≤1.5 × inter‐quantile‐range. Different letters indicate statistically significant differences (Wilcoxon signed‐rank test, FDR adjusted *p* < 0.05). Spearman correlation (*p* < 0.05) of risk index with environmental (D) and anthropogenic (E) factors.

Although the risk indices of vertebrate viruses were similar across host groups and family levels, human‐related viruses had slightly higher risk indices than others (Figure S9) and the vertebrate viruses with the highest risk indices were human‐related, which may pose a greater risk to public health and deserve special concern. However, as for each sample, agricultural soils posed the highest risk of vertebrate viruses in both MG and MT samples (Figure 2A–C).

Further, we calculated the correlations between values of risk index with environmental and anthropogenic factors to identify potential drivers of sample risks (Figure 2D,E). The effect size results revealed large contributions of environmental and anthropogenic factors to risk index (Table S6). These results indicate agricultural intensification may create favorable conditions for virus persistence and transmission. Risk index significantly positively correlates with land use change, human development, and population density—factors that increase host density and contact rates while creating novel spillover routes through practices like manure application and irrigation [18]. Frequent agricultural disturbances can also reshape environments, enhancing spillover risks [6]. Additionally, climate effects may amplify these risks, with positive correlations between viral risk and precipitation/temperature. Agricultural lands experience more extreme temperature fluctuations and altered hydrology, increasing spillover risk and cross‐species transmission risk, particularly during extreme weather events (e.g., heatwaves and heavy rainfall) and long‐term warming [19]. Agricultural practices can also reduce soil health factors that naturally suppress viruses. Risk index negatively correlates with soil fertility properties (e.g., cation exchange capacity and organic carbon). Intensive agriculture can decrease soil organic matter and disrupt soil structure, reducing virus‐binding capacity and increasing viral mobility [20]. These interconnected mechanisms create compound effects where agriculture increases viral inputs while reducing natural suppression factors. These effects would likely intensify with ongoing global changes like drought, global warming, and soil erosion [8], which could be strongly affected by agricultural activities directly and indirectly. This synthesis of current research with our results points to the need for holistic approaches to managing the potential health risk of vertebrate viruses by integrating global climate actions with agricultural practices.

## CONCLUSION

Emerging pandemics, none of which is an isolated event, reveal global vulnerability to vertebrate viruses. Surveillance is therefore crucial. Our study screened a large‐scale soil metagenome and metatranscriptome data set to expand the monitored vertebrate virus spectrum across global soils. Despite limitations in the vertebrate virus database size, potential database bias (e.g., overrepresentation of human and livestock viruses), soil type and sampling depth variations, geographic range of samples, temporal resolution, and land use intensity, our study emphasizes that soil is a significant mediator of vertebrate viruses and reveals that their health risks are aggravated in agricultural soils, offering a new perspective on comprehension of risks associated with agricultural expansion and intensification. Future studies combining metagenomic approaches with assessments of viral infectivity, temporal/land‐use experimental designs, identification of transmission routes, and ecological modeling will be crucial for better understanding the public health implications of these findings.

## METHODS

Detailed procedures for sample/data collection, sequencing protocol, and bioinformatic and statistical analysis approaches are available in the Supporting Information.

## AUTHOR CONTRIBUTIONS

**Kankan Zhao**: Methodology; investigation; visualization; formal analysis; writing—original draft; writing—review and editing; data curation. **Yiling Wang**: Methodology; writing—review and editing; data curation; formal analysis. **Ran Xue**: Formal analysis; methodology; writing—review and editing; data curation. **Xingmei Liu**: Conceptualization; investigation; funding acquisition; writing—review and editing; visualization; supervision. **Bin Ma**: Conceptualization; investigation; writing—review and editing; funding acquisition; visualization; supervision. **Jianming Xu**: Supervision; writing—review and editing; funding acquisition; conceptualization.

## CONFLICT OF INTEREST STATEMENT

The authors declare no conflicts of interest.

## ETHICS STATEMENT

No animals or humans were involved in this study.

## Supporting information


**Figure S1.** Geographic distribution of soil samples.
**Figure S2.** Overview of vertebrate viruses detected in samples.
**Figure S3.** Comparison of BLASTn to Kraken2 on the performance of vertebrate virus read identification.
**Figure S4.** Accumulation curves.
**Figure S5.** Relationships between the number of vertebrate viruses and detections in metagenome and metatranscriptome samples.
**Figure S6.** Estimating vertebrate virus richness in soils.
**Figure S7.** Richness and total abundance of metagenome and metatranscriptome samples across ecosystems.
**Figure S8.** Risk index of vertebrate viruses in metagenome and metatranscriptome samples.

**Table S1.** Samples used in this study.
**Table S2.** Vertebrate virus database.
**Table S3.** Vertebrate viruses detected in this study.
**Table S4.** Samples detected vertebrate viruses.
**Table S5.** Samples used in performance evaluation of BLASTn.
**Table S6.** Effect sizes of ecosystems, environmental and anthropogenic factors on risk index.
**Table S7.** Environmental and anthropogenic factors used in this study.

## Data Availability

The data that support the findings of this study are openly available in NCBI SRA at https://www.ncbi.nlm.nih.gov/, reference number PRJNA983538. All in‐house sequence data have been deposited in the NCBI SRA under BioProject accession number PRJNA983538 (https://www.ncbi.nlm.nih.gov/bioproject/PRJNA983538/). Publicly available metagenome and metatranscriptome samples are listed in Table S1. Publicly available environmental and anthropogenic factors are listed in Table S7. The data and scripts used are saved in GitHub https://github.com/SysMicro/Zhao2025iMeta. Supplementary materials (methods, figures, tables, graphical abstract, slides, videos, Chinese translated version, and update materials) may be found in the online DOI or iMeta Science http://www.imeta.science/.
