# Supplementary material for: Agriculture increases potential health risks of vertebrate viruses in soils
Source: Imeta. 2025 Apr 16;4(3):e70034. doi: 10.1002/imt2.70034 (PMC12130566; doi:10.1002/imt2.70034)
Supplement: Supplementary file 1 — Figure S1. Geographic distribution of soil samples. Figure S2. Overview of vertebrate viruses detected in samples. Figure S3. Comparison of BLASTn to Kraken2 on the performance of vertebrate virus read identification. Figure S4. Accumulation curves. Figure S5. Relationships between the number of vertebrate viruses and detections in metagenome and metatranscriptome samples. Figure S6. Estimating vertebrate virus richness in soils. Figure S7. Richness and total abundance of metagenome and metatranscriptome samples across ecosystems. Figure S8. Risk index of vertebrate viruses in metagenome and metatranscriptome samples. [file IMT2-4-e70034-s001.docx]

Supplementary Information to

**Agriculture** **increases potential health risks of vertebrate viruses in soils**

**Running title:** Agriculture increases soil health risks

Kankan Zhao^1,2^, Yiling Wang^1,2^, Ran Xue^1,2,3^, Xingmei Liu^1,2*^, Bin Ma^1,2,3*^, Jianming Xu^1,2^

^1^Institute of Soil and Water Resources and Environmental Science, College of Environmental and Resource Sciences, Zhejiang University, Hangzhou 310058, China

^2^Zhejiang Provincial Key Laboratory of Agricultural Resources and Environment, Zhejiang University, Hangzhou 310058, China

^3^ZJU-Hangzhou Global Scientific and Technological Innovation Center, Hangzhou 311200, China

**^*^**Correspondence: [xmliu@zju.edu.cn](mailto:xmliu@zju.edu.cn) (Xingmei Liu), [bma@zju.edu.cn](mailto:bma@zju.edu.cn) (Bin Ma)

**METHODS**

**Sampling and sequencing**

In total, 3049 soil metagenome (629 in-house samples and 2420 publicly available samples) and 1441 metatranscriptome samples (108 in-house samples and 1333 publicly available samples) were used in this study. In-house metagenome samples were sampled and sequenced from 2018 to 2021 using a uniform protocol as described before [1]. Briefly, samples were transported on ice or dry ice and were stored at -80 °C until DNA extraction. DNA was extracted with MP FastDNA SPIN Kits for soil (MP Biomedicals, Solon, OH, USA) as per manufacturer’s instructions. In-house metatranscriptome samples were sampled and preserved in 2020 and 2021 as described previously [2,3]. Soil total RNA was extracted with RNA PowerSoil Total RNA Isolation Kit (MoBio, CA, USA) and was depleted of rRNA using the Ribo-Zero rRNA Removal Kit (Illumina, CA, USA) according to the manufacturer’s protocols. Then, cDNA was synthesized by TruSeq Stranded mRNA LT Sample Prep Kit (Illumina, CA, USA). DNA and cDNA were sequenced on Illumina HiSeq 4000 or Illumina NovaSeq 6000 sequencing platform (Illumina, CA, USA). Publicly available metagenome and metatranscriptome samples, which should be Illumina paired-end sequencing data (metatranscriptome samples should also be generated from RNA with rRNA depletion), were retrieved from Genome Sequence Archive (GSA), Sequence Read Archive (SRA), and European Sequence Archive (ENA) on September 2, 2021 and May 5, 2022, respectively (Table S1).

**Vertebrate virus detection**

Raw data were trimmed and filtered with Trimmomatic (v0.39) [4] (length cut-off of 50 bp, quality cut-off of 20) as before [5], and metatranscriptomic reads mapping to rRNA were removed by SortMeRNA (v4.3.3) [6]. Due to the dominance of prokaryotic reads in metagenome and metatranscriptome data, it is hard to fully reconstruct virus genomes from the comparatively low abundance of vertebrate virus with incomplete coverage [7]. Therefore, we only focused on known vertebrate viruses. We used BLASTn program (v2.9.0) aligning metagenome and metatranscriptome data against a manually curated database of vertebrate (excluding aquatic ones) viruses to find hits with an optimization criterion (*e*-value cutoff of 1  ×  10^−5^, at least 95% identity over 75 nt alignment), more stringent than those used in some previous studies [8,9]. While this stringency may have reduced overall detection sensitivity, it strengthened the reliability of our findings regarding the presence and distribution patterns of vertebrate viruses across different ecosystems. Briefly, 2917 vertebrate virus genomes (excluding retroviruses) were manually collected from NCBI RefSeq Database (September 2, 2021), including 700 dsDNA genomes, 486 ssDNA genomes, 413 dsRNA genomes, 1291 ssRNA genomes, and 27 genomes with unknown molecule types (Table S2). In metagenome samples, only dsDNA genomes (700 dsDNA genomes) were kept for downstream analysis. The host information of viruses was found in Virus-Host DB (https://www.genome.jp/virushostdb/) according viral RefSeq ID.

We also employed Kraken2 (v2.1.2) [10] against NCBI RefSeq Database to identify vertebrate virus reads in 100 metagenome samples and 100 metatranscriptome samples (Table S5) to evaluate the performance of BLASTn. We then retrieved the vertebrate virus reads with Bracken (v2.8) [11]. Particularly, vertebrate virus read would be deleted if it was solely detected in a sample by Kraken2 but not by BLASTn.

Abundance of a vertebrate virus ($\alpha_{i}$) was calculated as the number of hits ($\varepsilon_{i}$) to vertebrate virus genomes normalized to sample size (*ω*, Gb) [8].

$$\begin{aligned} \alpha_{i}= \frac{\varepsilon_{i}}{\omega}\#\left( 1 \right) \end{aligned}$$

**Risk index assessment**

For each vertebrate virus *i*, risk index represents its ability to transfer from soils to vertebrates [12] and is calculated as

$$\begin{aligned} \mu_{i}= \bar{\alpha_{i}} \times\lambda_{i} \times\gamma_{i}\#\left( 2 \right) \end{aligned}$$

where $\mu_{i}$ is risk index of vertebrate virus *i*, $\bar{\alpha_{i}}$ is its average abundance, $\lambda_{i}$ is its occupancy (the ratio of samples detected vertebrate virus *i* to total samples), and $\gamma_{i}$ is its hazard classification formulated by the National Health Commission of the People’s Republic of China (https://zwfw.nhc.gov.cn/kzx/zcfg/gzbxbywswsyhdsp_242/202007/t20200716_1677.html, in Chinese; translated in Table S3).

For each sample, sample richness was also considered in the calculation and risk index is calculated as

$$\begin{aligned} \mu= \beta\times\sum_{i=1}^{n} (\alpha_{i} \times\mu_{i})\#\left( 3 \right) \end{aligned}$$

where $\mu$is risk index of a sample, $\beta$ is sample richness, $\alpha_{i}$ is abundance of vertebrate virus *i* in the sample, and $\mu_{i}$ is its risk index calculated using Eq. (2).

A total of 80 global layers were used to calculate the correlation between risk index and environmental and anthropogenic factors (Table S7) as described before [13]. Briefly, the information was converted into a unified pixel grid in EPSG:4326 (WGS84) at a 0.01-degree resolution using a nearest neighbor method. Then sample metadata (environmental and anthropogenic factors) was retrieved by their geographic locations and ecosystem information was manually adjusted according to the sample description in the sequencing data repository (Table S4).

**Statistical analysis**

All data analyses were conducted in R (R Core Team, 2019). Vertebrate virus richness was estimated by *prestonfit* and *prestondistr* functions of R package *vegan* [14] and *Diversity* function of R package *SpadeR* [15]. Significant differences between groups were evaluated by Wilcoxon rank-sum tests and adjusted with false discovery rate (FDR) control. Effect sizes were calculated by Evident (v0.4.0) [16], numeric data were transformed to categories using quartiles; we removed all categories that contained < 1% of all samples.

**REFERENCES**

1. Ma, Bin, Caiyu Lu, Yiling Wang, Jingwen Yu, Kankan Zhao, Ran Xue, Hao Ren, et al. 2023. “A genomic catalogue of soil microbiomes boosts mining of biodiversity and genetic resources.” *Nature Communications* 14: 7318. <https://doi.org/10.1038/s41467-023-43000-z>

2. Zhao, Kankan, Bin Ma, Yan Xu, Erinne Stirling, and Jianming Xu. 2021. “Light exposure mediates circadian rhythms of rhizosphere microbial communities.” *The ISME Journal* 15: 2655–2664. <https://doi.org/10.1038/s41396-021-00957-3>

3. Zhao, Kankan, Haodan Yu, Ran Xue, Erinne Stirling, Yiling Wang, Jianming Xu, and Bin Ma. 2022. “The only constant is change: Endogenous circadian rhythms of soil microbial activities.” *Soil Biology and Biochemistry* 173: 108805. <https://doi.org/10.1016/j.soilbio.2022.108805>

4. Bolger, Anthony M., Marc Lohse, and Bjoern Usadel. 2014. “Trimmomatic: a flexible trimmer for Illumina sequence data.” *Bioinformatics* 30: 2114–2120. <https://doi.org/10.1093/bioinformatics/btu170>

5. Wang, Binhao, Jianming Xu, Yiling Wang, Erinne Stirling, Kankan Zhao, Caiyu Lu, Xiangfeng Tan, et al. 2023. “Tackling soil ARG-carrying pathogens with global-scale metagenomics.” *Advanced Science* 10: 2301980. <https://doi.org/10.1002/advs.202301980>

6. Kopylova, Evguenia, Laurent Noé, and Hélène Touzet. 2012. “SortMeRNA: fast and accurate filtering of ribosomal RNAs in metatranscriptomic data.” *Bioinformatics* 28: 3211–3217. <https://doi.org/10.1093/bioinformatics/bts611>

7. Hill, Sarah C., Sarah François, Julien Thézé, Adrian L. Smith, Peter Simmonds, Christopher M. Perrins, Lia van der Hoek, and Oliver G. Pybus. 2023. “Impact of host age on viral and bacterial communities in a waterbird population.” *The ISME Journal* 17: 215–226. <https://doi.org/10.1038/s41396-022-01334-4>

8. Aziz, Ramy K., Bhakti Dwivedi, Sajia Akhter, Mya Breitbart, and Robert A. Edwards. 2015. “Multidimensional metrics for estimating phage abundance, distribution, gene density, and sequence coverage in metagenomes.” *Frontiers in Microbiology* 6: 381. <https://doi.org/10.3389/fmicb.2015.00381>

9. Dutilh, Bas E., Noriko Cassman, Katelyn McNair, Savannah E. Sanchez, Genivaldo G. Z. Silva, Lance Boling, Jeremy J. Barr, et al. 2014. “A highly abundant bacteriophage discovered in the unknown sequences of human faecal metagenomes.” *Nature Communications* 5: 4498. <https://doi.org/10.1038/ncomms5498>

10. Wood, Derrick E., Jennifer Lu, and Ben Langmead. 2019. “Improved metagenomic analysis with Kraken 2.” *Genome Biology* 20: 257. <https://doi.org/10.1186/s13059-019-1891-0>

11. Lu, Jennifer, Florian P. Breitwieser, Peter Thielen, and Steven L. Salzberg. 2017. “Bracken: estimating species abundance in metagenomics data.” *PeerJ Computer Science* 3: e104. <https://doi.org/10.7717/peerj-cs.104>

12. Zhang, Zhenyan, Qi Zhang, Tingzhang Wang, Nuohan Xu, Tao Lu, Wenjie Hong, Josep Penuelas, et al. 2022. “Assessment of global health risk of antibiotic resistance genes.” *Nature Communications* 13: 1553. <https://doi.org/10.1038/s41467-022-29283-8>

13. Ma, Bin, Yiling Wang, Zhao Kankan, Stirling Erinne, Lv Xiaofei, Yu Yijun, Hu Lingfei, et al. 2024. “Biogeographic patterns and drivers of soil viromes.” *Nature Ecology & Evolution* 8: 717–728. <https://doi.org/10.1038/s41559-024-02347-2>

14. Dixon, Philip. 2003. “VEGAN, a package of R functions for community ecology.” *Journal of Vegetation Science* 14: 927–930. <https://doi.org/10.1111/j.1654-1103.2003.tb02228.x>

15. Chao, Anne, K. H. Ma, T. C. Hsieh, and Chun-Huo Chiu. 2016. “SpadeR: species-richness prediction and diversity estimation with R.” *R package version 0.1.1*. <https://cran.r-project.org/web/packages/SpadeR>

16. Rahman, Gibraan, Daniel McDonald, Antonio Gonzalez, Yoshiki Vázquez-Baeza, Lingjing Jiang, Climent Casals-Pascual, Daniel Hakim, et al. 2023. “Determination of effect sizes for power analysis for microbiome studies using large microbiome databases.” *Genes* 14: 1239. <https://doi.org/10.3390/genes14061239>


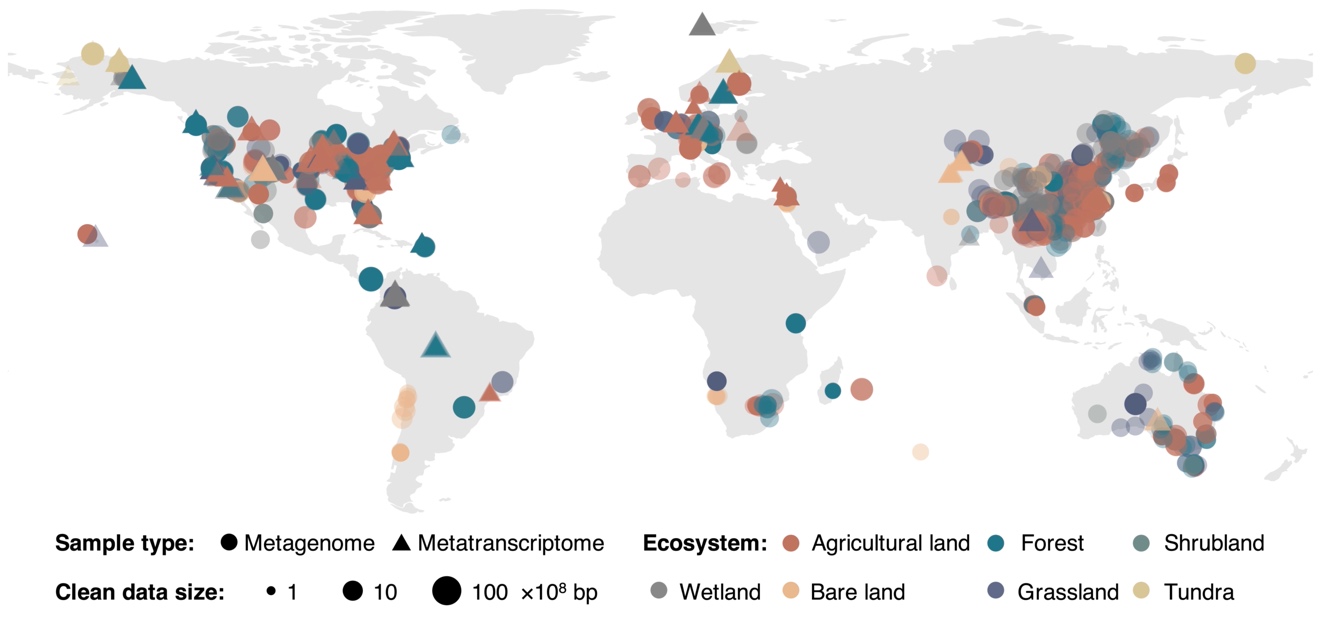


**Figure S1 Geographic distribution of soil samples.** Samples were shaped by sample type, colored by ecosystems, and sized by clean data size.

**
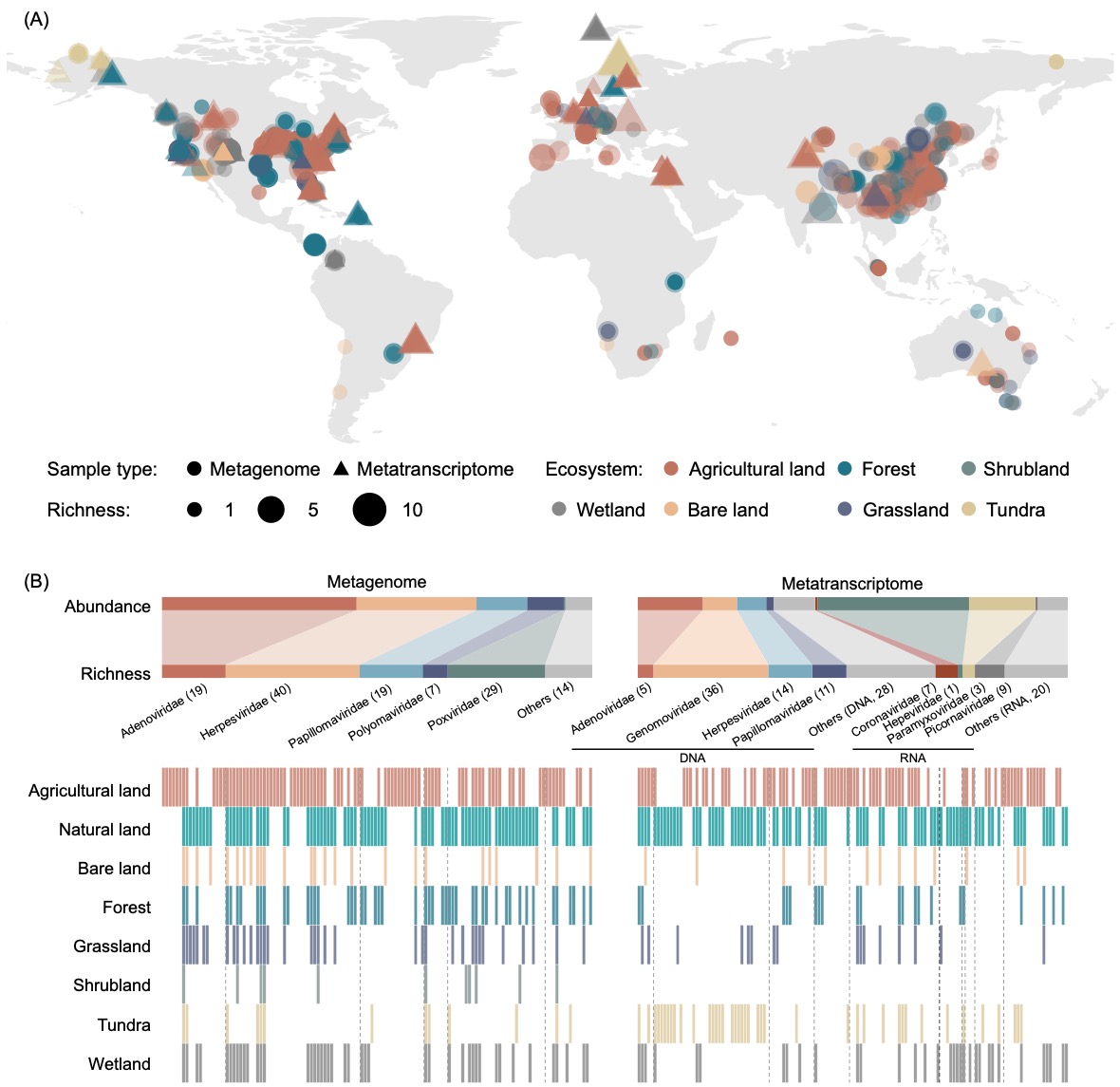
**

**Figure S2 Overview of vertebrate viruses detected in samples.** (A) Geographic prevalence of metagenome (circles) and metatranscriptome (triangles) samples (colored by ecosystems and sized by richness) detected vertebrate viruses. (B) The upper panels show the proportions of vertebrate viruses (colored by families of vertebrate viruses) in abundance and richness among metagenome and metatranscriptome samples. The lower panels show ecosystems that detected vertebrate viruses corresponding to the upper panels (colors represent viruses that existed in the soils of certain ecosystems; non-agricultural ecosystems were grouped into “Natural land”).

**
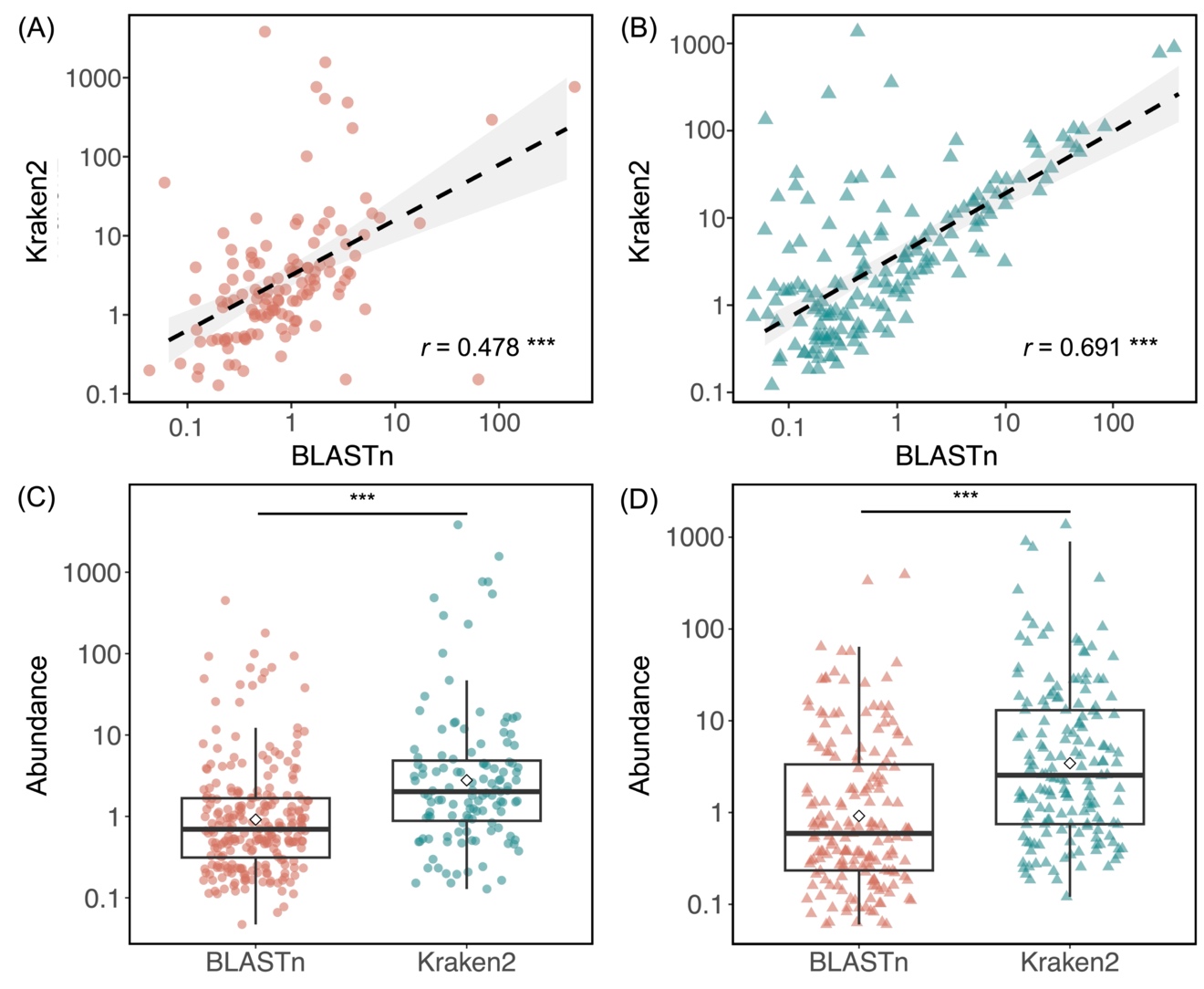
**

**Figure S3 Comparison of BLASTn to Kraken2 on the performance of vertebrate virus read identification.** Relationships between abundance of BLASTn and Kraken2 approaches in metagenome (A) and metatranscriptome (B) samples. Abundance comparison between BLASTn and Kraken2 approaches in metagenome (C) and metatranscriptome (D) samples. The dash lines are regression lines, and the shaded area is the 95% confidence interval. Boxes are vertically bounded by the 1st and 3rd quartiles, center lines are medians, diamonds are means and whiskers extend to ≤ 1.5 × inter-quantile-range. Shapes represent sample types, *r* represents Pearson correlation coefficient, and symbol *** represents *p* < 0.001.


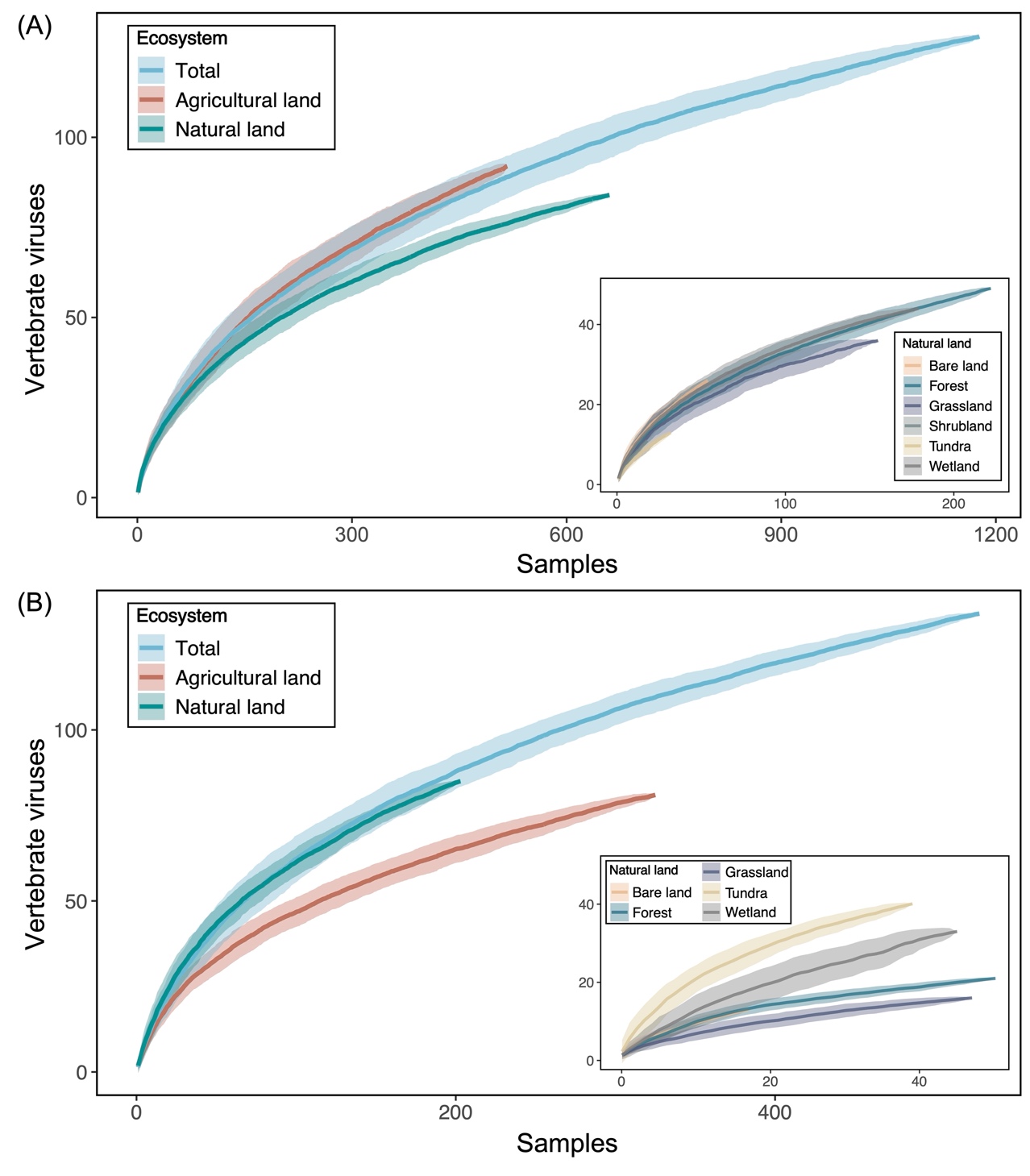


**Figure S4 Accumulation curves.** Accumulation curves for total samples and subsamples separated by ecosystems of metagenome (A) and metatranscriptome (B) samples. Colors represent ecosystems and non-agricultural ecosystems were grouped into “Natural land”.


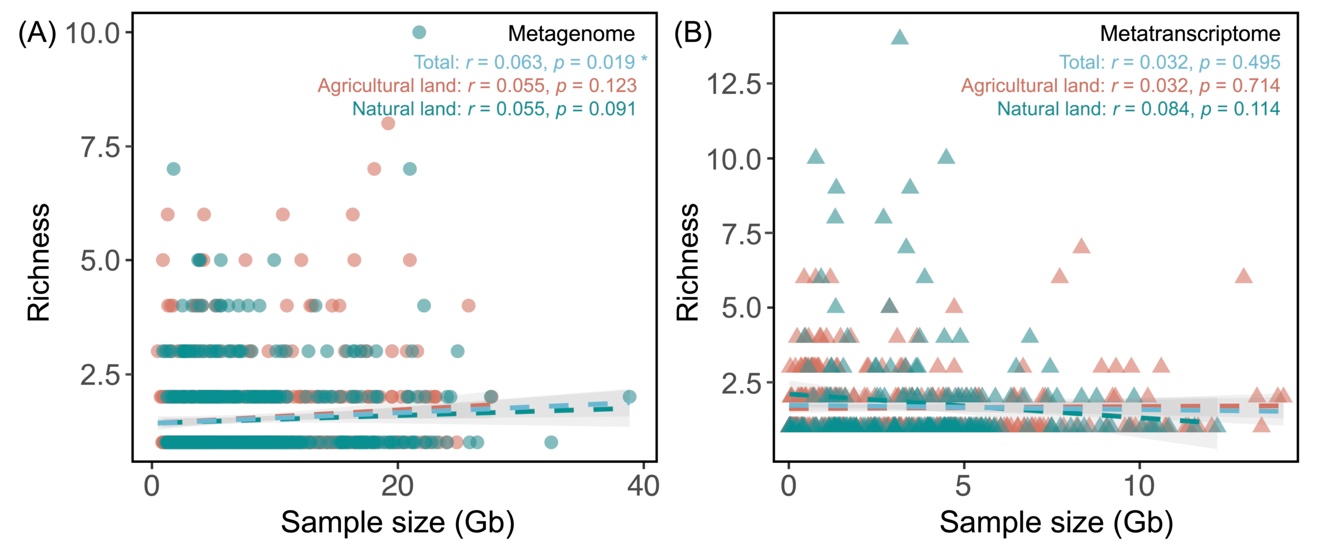
 **Figure S5 Relationships between richness and sample size of metagenome (A) and metatranscriptome (B) samples.** The dash lines are regression lines, the shaded area is the 95% confidence interval, the colors represent ecosystems, and non-agricultural ecosystems were grouped into “Natural land”. r represents Pearson correlation coefficient, and symbol * represents *p* < 0.05.

**
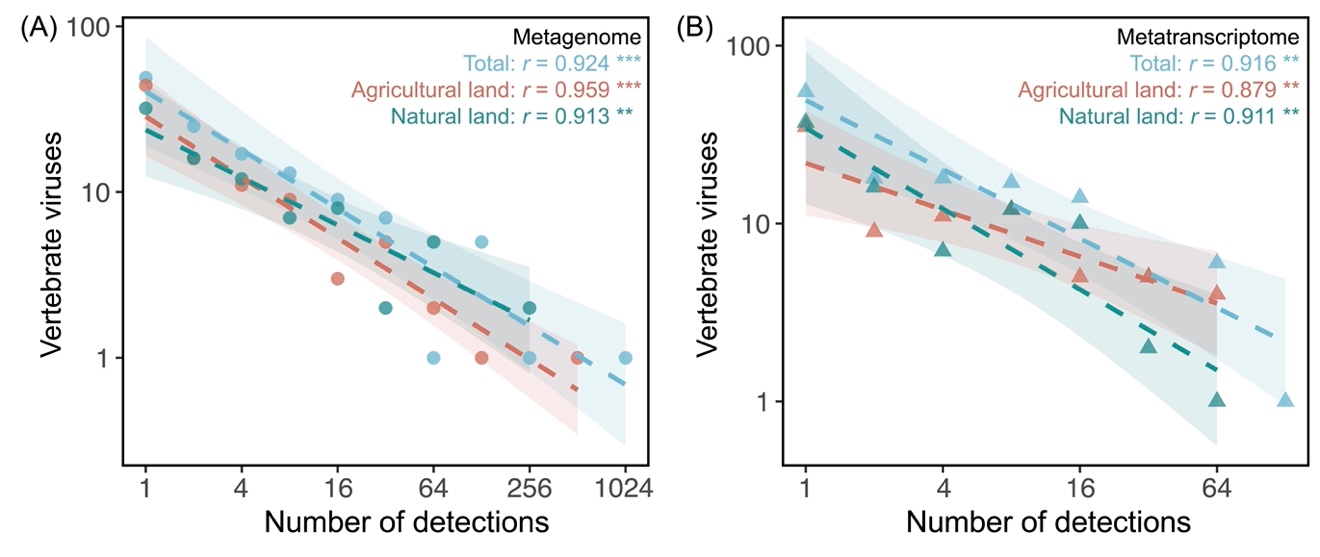
**

**Figure S6 Relationships between the number of vertebrate viruses and detections in metagenome (A) and metatranscriptome (B) samples.** The dash lines are regression lines, the shaded area is the 95% confidence interval, the colors represent ecosystems, *r* represents Pearson correlation coefficient, significance levels of each symbol are ****p* < 0.001 and ***p* < 0.01.

**
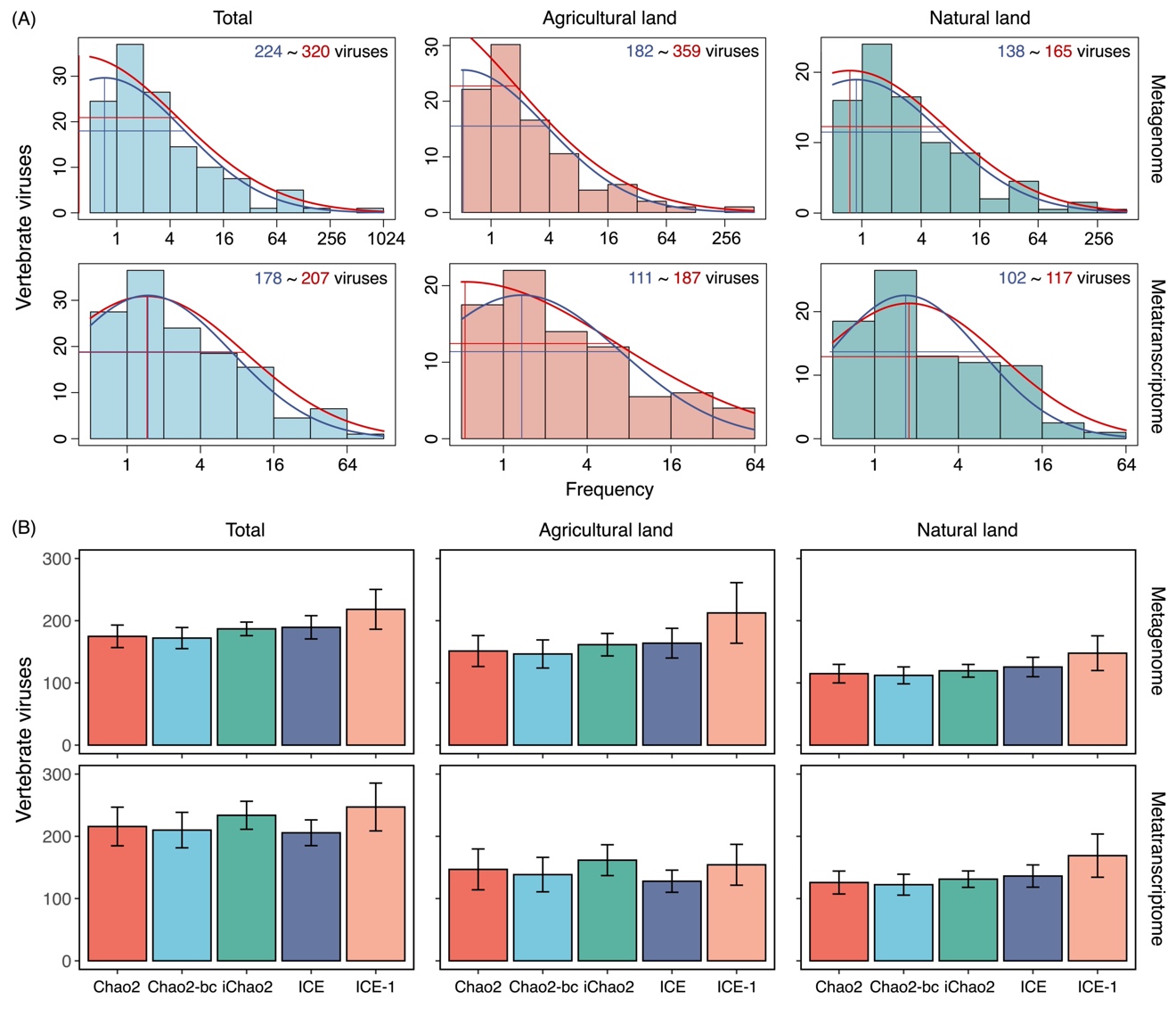
**

**Figure S7 Estimating vertebrate virus richness in soils.** (A) Estimating vertebrate virus richness with truncated Preston log-normal model. Blue line grouped frequencies of vertebrate viruses into doubling octave classes and fitted truncated Preston log-normal. The red line fitted the truncated Preston log-normal model without pooling the data into octaves. (B) Estimating vertebrate virus richness with Chao2, Chao2-bc, iChao2, ICE, and ICE-1 estimators. Error bars represent standard error.


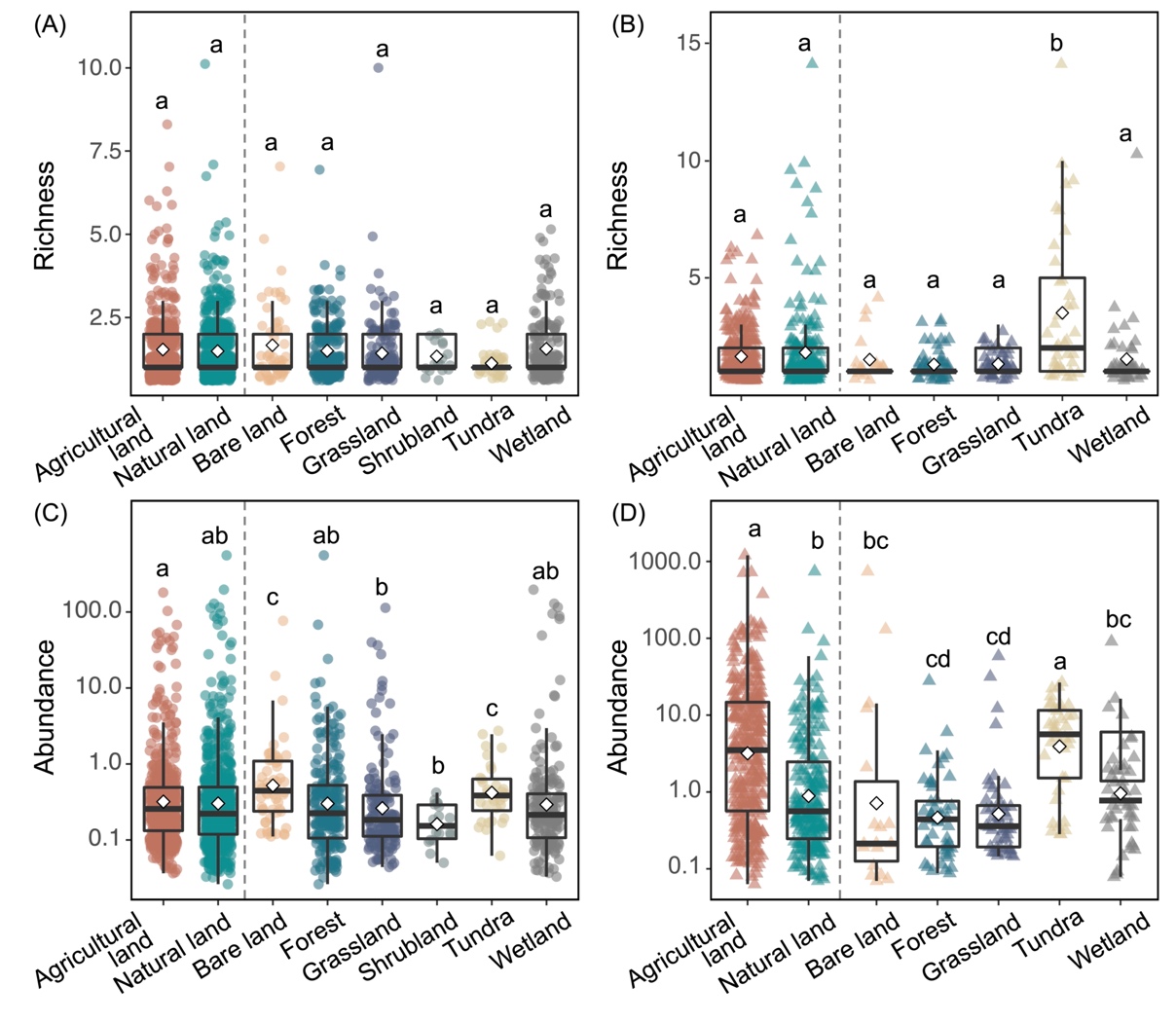


**Figure S8** **Richness and total abundance of metagenome (A and C) and metatranscriptome (B and D) samples across ecosystems.** Colors represent ecosystems, shapes represent sample types, boxes are vertically bounded by the 1st and 3rd quartiles, center lines are medians, diamonds are means and whiskers extend to ≤ 1.5 × inter-quantile-range. Different letters indicate statistically significant differences (Wilcoxon signed-rank test, FDR adjusted *p*  <  0.05).


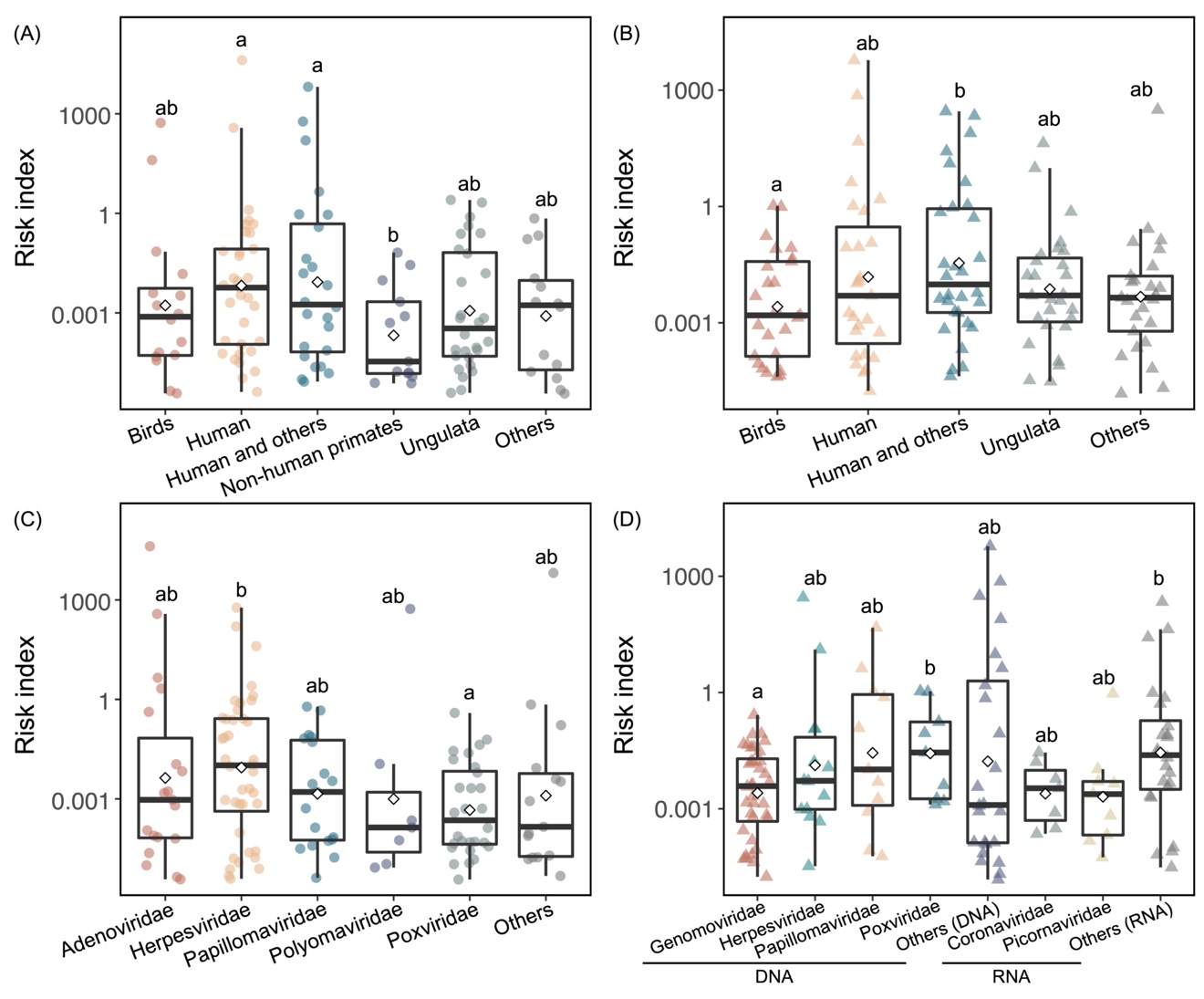


**Figure S9 Risk index of vertebrate viruses in metagenome and metatranscriptome samples.** Colors represent host groups (A and B) or families (C and D) of vertebrate viruses, shapes represent sample types, boxes are vertically bounded by the 1st and 3rd quartiles, center lines are medians, diamonds are means and whiskers extend to ≤ 1.5 × inter-quantile-range. Different letters indicate statistically significant differences (Wilcoxon signed-rank test, FDR adjusted *p*  <  0.05).
